# Comparing reactive and memory-one strategies of direct reciprocity

**DOI:** 10.1038/srep25676

**Published:** 2016-05-10

**Authors:** Seung Ki Baek, Hyeong-Chai Jeong, Christian Hilbe, Martin A. Nowak

**Affiliations:** 1 Department of Physics, Pukyong National University, Busan 48513, Korea; 2 Department of Physics and Astronomy, Sejong University, Seoul 05006, Korea; 3 IST Austria, Am Campus 1, 3400 Klosterneuburg, Austria; 4 Program for Evolutionary Dynamics, Department of Mathematics, Department of Organismic and Evolutionary Biology, Harvard University, Cambridge, MA 02138, United States of America

## Abstract

Direct reciprocity is a mechanism for the evolution of cooperation based on repeated interactions. When individuals meet repeatedly, they can use conditional strategies to enforce cooperative outcomes that would not be feasible in one-shot social dilemmas. Direct reciprocity requires that individuals keep track of their past interactions and find the right response. However, there are natural bounds on strategic complexity: Humans find it difficult to remember past interactions accurately, especially over long timespans. Given these limitations, it is natural to ask how complex strategies need to be for cooperation to evolve. Here, we study stochastic evolutionary game dynamics in finite populations to systematically compare the evolutionary performance of reactive strategies, which only respond to the co-player’s previous move, and memory-one strategies, which take into account the own and the co-player’s previous move. In both cases, we compare deterministic strategy and stochastic strategy spaces. For reactive strategies and small costs, we find that stochasticity benefits cooperation, because it allows for generous-tit-for-tat. For memory one strategies and small costs, we find that stochasticity does not increase the propensity for cooperation, because the deterministic rule of win-stay, lose-shift works best. For memory one strategies and large costs, however, stochasticity can augment cooperation.

Direct reciprocity, the propensity to return cooperative acts of others, is one of the major mechanisms to establish cooperation^1^,^2^,^3^. The theory of reciprocity has allowed us to understand under which conditions “a shadow of the future” can help individuals to forego individual short-run benefits in favour of mutually beneficial long-run relationships^4^,^5^,^6^,^7^,^8^,^9^,^10^,^11^,^12^,^13^. Although reciprocal relationships also seem to be at work in several animal species^14^,^15^,^16^, they play a particular role for human interactions^17^. Because almost all our social interactions occur repeatedly, reciprocity considerations may have played an important role for the evolution of social heuristics^18^,^19^, which in turn helps to understand why we also cooperate with strangers^20^, sometimes even without considering the resulting costs to ourselves^21^.

To model the emergence of direct reciprocity, researchers often use the example of the iterated prisoner’s dilemma. In this game, two players can decide repeatedly whether to cooperate or to defect. While mutual cooperation is optimal from a group perspective, players may feel a temptation to defect at the expense of the co-player. Strategies for the repeated prisoner’s dilemma can become arbitrarily complex—sophisticated players may use the whole past history of play when making the decision whether to cooperate in the next round. In practice, however, several experiments suggest that the complexity of human strategies is restricted. For example, Stevens *et al*.^22^ have shown that subjects have problems to remember their co-players’ past decisions accurately, especially if they need to keep track of several co-players or multiple rounds. Similarly, the research of Wedekind and Milinski^23^,^24^ suggests that there is a trade-off between having a sophisticated strategy in the prisoner’s dilemma and performing well in a second unrelated task. In addition, recent studies in behavioural economics have found that most of the strategies employed by human subjects are well described by simple strategies that only depend on the last interaction^25^,^26^,^27^,^28^,^29^, although there are also other factors such as the average fraction of past cooperative acts^30^,^31^. Given that there are such constraints on the complexity of strategies, can we still expect cooperation to evolve? And how complex do the players’ strategies need to be in order to allow for substantial cooperation?

Herein, we approach this question by comparing the evolving cooperation rates for different strategy spaces for the repeated prisoner’s dilemma. The considered strategy spaces differ along two dimensions of complexity. The first dimension is the input that they require: whereas reactive strategies (or memory-1/2 strategies) only require information about the co-player’s previous move^32^,^33^,^34^, memory-one strategies additionally need to take one’s own move into account^35^. The set of reactive strategies is a reasonable and conventional choice to define a subset of memory-one strategies, because a player’s payoff crucially depends on the co-player’s move in the prisoner’s dilemma. The second dimension is the strategy’s stochasticity. Here, we distinguish strategies that respond to past outcomes in a deterministic fashion, and strategies that prescribe to randomize. Overall, these two independent dimensions of complexity lead to four different strategy classes.

To assess whether a given strategy class is favourable to the evolution of cooperation, we consider the Moran process in a finite population of players^36^. Individuals can choose freely among the available strategies, and over time they learn to switch to strategies that yield a higher payoff. By assuming that mutations are sufficiently rare, we can use the framework of Fudenberg & Imhof 37 to calculate how often players use each of the available strategies in the long run^38^. This in turn allows us to calculate the evolving cooperation rates for each of the four strategy classes, as explained in more detail in the next section. Our results suggest that strategies with larger memory are typically beneficial for the evolution of cooperation, whereas the strategies' stochasticity can sometimes have a detrimental effect.

## Model and Methods

It is common to consider two levels when modelling the evolutionary dynamics of repeated games. The first level focuses on the repeated game itself. At this level, we look at a single instance of the repeated game and we calculate how the players’ strategies determine the resulting cooperation rates and average payoffs. The second level describes the population dynamics. Here, we look at a whole population of players. Each player is equipped with a strategy for how to play the repeated game. The abundance of a given strategy within the population may change over time, because strategies that lead to a high payoff are expected to spread (either due to reproduction of successful individuals, or due to imitation and cultural learning). At the population level, we are interested in how often a strategy will be used in the long run, and what the resulting average cooperation rate is. In the following, we describe these two levels in more detail.

### Game dynamics of the repeated prisoner’s dilemma

In the prisoner’s dilemma, two individuals decide simultaneously whether to cooperate (*C*) or to defect (*D*). A player who cooperates pays a cost *c* > 0 to provide a benefit *b* > *c* for the co-player. Thus, a cooperator either gets *b* − *c* (if the co-player cooperates as well) or −*c* (if the co-player defects). On the other hand, a defector either gets *b* (if the co-player cooperates) or 0 (if the co-player defects). To reduce the number of free parameters, we can set *b*: = 1 and we let *c* vary between 0 < *c* < 1. Moreover, to avoid negative payoffs, we add the constant *c* to all payoffs. Under these assumptions, the payoff matrix of the prisoner’s dilemma takes the form


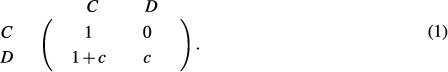


Because *c* < 1, both players prefer mutual cooperation over mutual defection; however, since *c* > 0, each individual is tempted to play *D* irrespective of the co-player's action. If the prisoner’s dilemma is played in a well-mixed population, evolution favours defection.

The question of evolutionary strategy selection becomes more interesting when individuals have the option to reciprocate past actions in the future. To model such repeated interactions, we consider two individuals who play the game (1) for infinitely many rounds. Strategies for such repeated games need to prescribe an action for any possible history of previous play, and they can become arbitrarily complex. To facilitate an evolutionary analysis, we assume herein that individuals at most make use of simple memory-one strategies. That is, their behaviour in any given round may only depend on the outcome of the previous round. Memory-one strategies can be written as a 4-tuple, **p** = (*p*_*CC*_, *p*_*CD*_, *p*_*DC*_, *p*_*DD*_). The entries *p*_*ij*_ correspond to the player’s probability to cooperate in the next round, given that the focal player's previous action was *i* and that the co-player’s action was *j*. We assume that players only have imperfect control over their actions, such that they mis-implement their intended action with some small probability *ε* > 0^5^,^39^. Under this assumption, the player's effective strategy becomes **p′** = (1 − *ε*)**p** + *ε*(1 − **p**).

When both players apply memory-one strategies **p** and **q**, respectively, then the dynamics of the repeated prisoner’s dilemma takes the form of a Markov chain with four possible states *CC*, *CD*, *DC*, *DD* (the possible outcomes of each round). The transition matrix of this Markov chain is given by


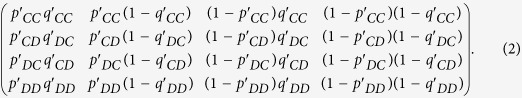


Due to the assumption of errors, all entries of this transition matrix are positive. Therefore, there exists a unique invariant distribution **v** = (*v*_*CC*_, *v*_*CD*_, *v*_*DC*_, *v*_*DD*_), representing the probability to find the two players in each of the four states over the course of the game. Given the invariant distribution **v**, we can calculate player 1’s payoff as *π*(**p**, **q**) = **v** · **h**_1_ and player 2’s payoff as *π*(**q**, **p**) = **v** · **h**_2_, with **h**_1_ = (1, 0, 1 + *c*, *c*) and **h**_2_ = (1, 1 + *c*, 0, *c*). Similarly, we can calculate the players’ average cooperation rate in the repeated game as *γ*(**p**, **q**) = *v*_*CC*_ + *v*_*CD*_ and *γ*(**q, p**) = *v*_*CC*_ + *v*_*DC*_. If the cooperation rate *γ*(**p, p**) of a strategy against itself converges to one as the error rate *ε* goes to zero, we call the strategy **p** a *self-cooperator* (see also ref. 40). Similarly, strategies for which the cooperation rate *γ*(**p, p**) approaches zero are called *self-defectors*.

We are interested in how the complexity of the strategy space affects the evolution of cooperation. To this end, we distinguish two dimensions of complexity. The first dimension is the input that the strategy takes into consideration. Players with a memory-1 strategy take the full outcome of the previous round into account, whereas players with a reactive strategy (or memory-1/2 strategy) only consider the co-player’s previous move (but not the own move). The second dimension is the strategy's stochasticity. Players with a deterministic strategy respond to past outcomes in a deterministic fashion, whereas players with a stochastic strategy may randomize between cooperation and defection. Combining these two dimensions, we end up with four different strategy spaces, as summarized in Table 1.

These four strategy spaces are partially ordered, 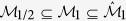 and 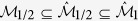 (there is no order between 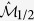 and 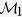). Examples of deterministic reactive strategies include *AllD* = (0, 0, 0, 0), *AllC* = (1, 1, 1, 1) and Tit-for-Tat, *TFT* = (1, 0, 1, 0). An example of a stochastic reactive strategy is generous Tit-for-Tat, *GTFT* = (1, 1 − *c*/*b*, 1, 1 − *c*/*b*) (see refs 41 and 42). Finally, as two examples of deterministic memory-one strategies which are not reactive, we mention the Grim Trigger strategy, *GT* = (1, 0, 0, 0), and Win-stay Lose-shift, *WSLS* = (1, 0, 0, 1). *GT* switches to relentless defection after any deviation from mutual cooperation; *WSLS*, on the other hand, sticks to an action if and only if it has been successful in the previous round^43^,^44^,^45^.

### Population dynamics

To describe the evolutionary dynamics on the population level, we use the Moran process^4^,^36^,^46^,^47^ in the limit of rare mutations^37^,^48^,^49^. That is, we consider a population of size *N*, and we suppose that new mutant strategies are sufficiently rare such that at any moment in time at most two different strategies are present in the population. If there are *i* individuals who adopt the strategy **p**, and *N* − *i* individuals who adopt the strategy **q**, the average payoffs for the two groups of players are


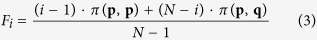



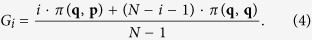


We assume that the fitness of a strategy is a linear function of its payoff. Specifically, if the fitness of the strategies **p** and **q** is denoted by *f*_*i*_ and *g*_*i*_, respectively, then


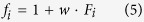



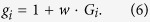


The constant terms on the right-hand side correspond to the player’s background fitness, and the parameter *w* is a measure for the strength of selection. When *w* → 0, payoffs become irrelevant, and both strategies have approximately equal fitness. We refer to this special case as the limit of weak selection.

The abundance of a strategy can change over time, depending on the strategy’s relative success. We consider a simple birth-death process. In each time step, one individual is randomly chosen for death, and its place is filled with the offspring of another individual, which is randomly chosen proportional to its fitness. That is, if 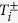 denotes the probability that the number of individuals with strategy **p** becomes *i* ± 1 after one time step, then we can calculate


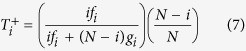



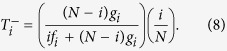


The quantities 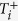 and 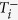 can be used to compute the probability that eventually the whole population will adopt strategy **p**^36^. In the special case that the population starts from a state in which only a single player applies **p**, this fixation probability *ρ* is given by


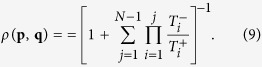


If there is no selection (i.e., if *w* = 0), the fixation probability for any mutant strategy **p** simplifies to *ρ*(**p**, **q**) = 1/*N*. For positive selection strength *w* > 0, we thus say that the mutant strategy **p** is advantageous, neutral, or disadvantageous if *ρ*(**p, q**) is larger, equal, or smaller than 1/*N*, respectively. Conversely, we say that the resident strategy **q** is evolutionary robust if there is no advantageous mutant strategy^40^,^50^.

For strategy spaces 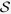 with finitely many strategies, 
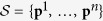
, we can use the above formula for the fixation probabilities to calculate the long-run abundance of each strategy. For sufficiently rare mutations, the evolutionary process can be described by a Markov chain with state space 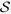, corresponding to the homogeneous populations in which everyone applies the same strategy (see ref. 37). The off-diagonal entries of the transition matrix *M* = (*m*_*jk*_) are given by *m*_*jk*_ = *ρ*(**p**^*k*^, **p **^*j*^)/(n − 1); starting in a population in which everyone uses strategy **p **^*j*^, the probability that the next mutant adopts strategy **p**^*k*^ is 1/(n − 1), and the probability that the mutant strategy reaches fixation is *ρ*(**p**^*k*^, **p**^*j*^). The diagonal entries of the transition matrix have the form 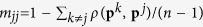, which can be interpreted as the probability that the next mutant strategy will go extinct. For any finite selection strength *w*, the stochastic transition matrix *M* has a unique invariant distribution **ξ** = (*ξ*_1_, …, *ξ*_*n*_). The entries of *ξ* represent the frequency with which each strategy is used in the selection-mutation equilibrium. Note that the exact value of the mutation rate is unimportant in calculating the invariant distribution as long as the transition matrix *M* is positive definite. Using this invariant distribution **ξ**, one can compute the average payoff in the population over time as


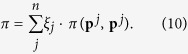


Similarly, one can compute the population’s average cooperation rate as


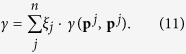


These two expressions average over all self-interactions of strategies, because in the rare-mutation limit the population is almost always homogeneous. The measure *γ* takes into account how much each strategy actually contributes to the cooperative behaviour of a population. A strategy’s contribution may not always be clear from its definition. For example, the strategy *GT* = (1, 0, 0, 0) is a self-defector (as any defection by mistake will cause it to respond with indefinite defection), whereas *WSLS* = (1, 0, 0, 1) is a self-cooperator, although the two strategies differ by just one bit.

When the strategy space is infinite (as for stochastic strategy spaces), we cannot apply the previous method directly. Instead, we use two different approximations. The first approach is to discretise the state spaces 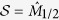 and 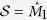. That is, instead of allowing for arbitrary conditional cooperation probabilities *p*_*ij*_ ∈ [0, 1], the probabilities are restricted to some finite grid *p*_*ij*_ = {0, 1/*m*, 2/*m*, …, 1}, where 1/*m* is the grid size. As our second approach, we use the method of Imhof & Nowak^51^. This method starts with an arbitrary resident strategy **p**^(0)^. This resident is then challenged by a single mutant with strategy **q**, with **q** being taken from a uniform distribution over the space of all memory-one strategies. If the mutant goes extinct, we define **p**^(1)^ = **p**^(0)^; otherwise, the mutant becomes the new resident and **p**^(1)^ = **q**. This elementary step is repeated for *t* iterations, leading to a sequence of successive resident populations (**p**^(0)^, **p**^(1)^, …, **p**^(*t*)^). Using this approach, we can calculate the average payoff of the population as 
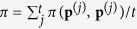
, and the average cooperation rate as 
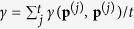
. As we will see, the two complementary approaches give similar results—provided that the grid size 1/*m* used for the first method is sufficiently small, and that the number of iterations *t* used for the second method is sufficiently large.

### Analytical methods in the limit of weak selection

In addition to the above numerical methods, one can use perturbative methods to compute exact strategy abundance in the limit of weak selection^52^,^53^. For a finite strategy space of size *n*, the assumption of weak selection implies that each strategy **p**^*i*^ is approximately played with probability 1/*n*, plus a deviation term that is proportional to


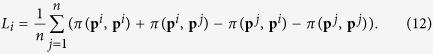


When *L*_*i*_ > 0, we say that the strategy **p**^*i*^ is favoured by selection. The analogous quantity for infinite strategy spaces (see also ref. 53) is given by


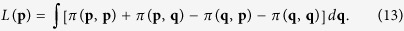


In this expression 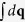 is the short-hand notation for the four-dimensional integral 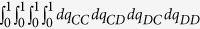, which in most cases needs to be computed numerically (see Appendix). By looking for maxima of *L*(**p**), we can determine the stochastic strategy that is most favoured by selection in the weak-selection limit.

## Results

In the following, we first discuss the dynamics in each of the four considered strategy spaces separately, and then we compare the resulting cooperation levels and average payoffs.

### Strategy dynamics among the deterministic reactive strategies

The space of deterministic reactive strategies 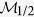 consists of the four strategies *AllD*, *AllC*, *TFT*, and the somewhat paradoxical Anti-Tit-for-Tat, *ATFT* = (0, 1, 0, 1), which cooperates if and only if the co-player was a defector in the previous round. For any set of parameters, we can use the methods explained in the previous section to calculate the fixation probability of a mutant with strategy **q** in an otherwise homogeneous population using strategy **p**.

**Figure 1:**
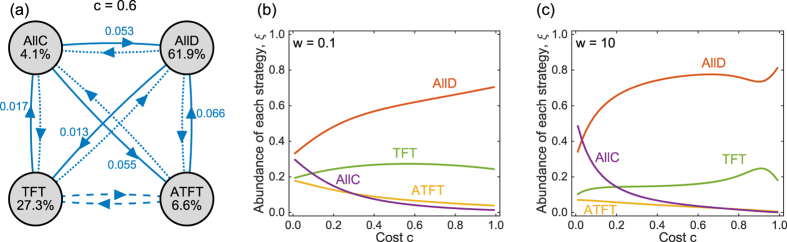
Evolutionary dynamics in the space of deterministic reactive strategies, 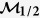. (**a**) Illustration of the dynamical process. Each grey circle represents a homogeneous population using one of the four possible strategies. Blue lines indicate whether a mutant strategy is advantageous (solid line), neutral (dashed line), or disadvantageous (dotted line). For advantageous mutants, the blue numbers show the mutant’s fixation probability according to Eq. (9). The graph suggests there are two likely paths for evolution: a short cycle from *AllD*, to *TFT* to *AllC* and back to *AllD*, or the longer cycle through *AllD*, *TFT*, *AllC*, *ATFT*, and back to *AllD* (in particular, eliminating the second cycle by removing *ATFT* from the strategy set would only lead to a minor modification of the general dynamics). The numbers within the grey circles give the abundance of each strategy according to the invariant distribution of the dynamical process; for the chosen parameters, *AllD* is the most abundant strategy. (**b,c**) show the abundance of each strategy depending on the cost of cooperation and for two different selection strengths *w* = 0.1 and *w* = 10. Other parameters: population size *N* = 100, error rate *ε* = 0.01, and in (**a**) *w* = 0.1.

Figure 1a illustrates this procedure in a population of size *N* = 100. If the resident population applies the strategy *AllD*, then neither *AllC* nor *ATFT* are advantageous. A single mutant player with strategy *TFT*, however, has a fixation probability *ρ* = 0.013 > 1/100 in an *AllD* population. *TFT* can invade because it cannot be exploited^54^,^55^,^56^,^57^: on average, a *TFT* player gets the mutual defection payoff *c* when matched with an *AllD*-opponent, but it gets (1 + *c*)/2 > *c* when interacting with a *TFT*-opponent. However, once *TFT* has reached fixation, a mutant adopting *AllC* can easily invade. *AllC* is more robust to errors—when two *TFT* players meet and one player defects by mistake, this can result in long and costly vendettas between the two players, whereas *AllC* players would not encounter that problem. But a homogeneous population of unconditional cooperators is quickly undermined by defectors, or by *ATFT* players (who themselves are typically replaced by defectors). Overall, we end up with an evolutionary cycle: cooperation can evolve starting from a population of defectors, but cooperation is not stable.

In the long run, most of the time is spent in a homogeneous *AllD* population (for the parameters used in Fig. 1a, the abundance of *AllD* is 61.9%). The reason for *AllD*’s predominance is its relative stability: it takes two *TFT* players to have a selective advantage in an *AllD* population (a single *TFT* player only obtains the same payoff *c* that the other *AllD* players receive). In contrast, it takes only one *AllC* player to have a selective advantage in a *TFT* population, and it takes only one *AllD* player to have an advantage in an *AllC* population. The dynamics within the space of deterministic reactive strategies is largely independent of the specific parameters being used. A numerical analysis shows that *AllD* remains the most abundant strategy in the selection-mutation equilibrium for both small (Fig. 1b) and large (Fig. 1c) selection strengths.

We can further confirm these numerical results by analytical means when we look at the limit of weak selection. For the space 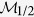, the linear coefficients *L*_*i*_ according to Eq. (12) simplify to


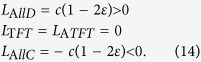


Thus, when selection is weak, *AllD* is the most abundant strategy for all values of *c*.

### Strategy dynamics among the deterministic memory-one strategies

**Figure 2:**
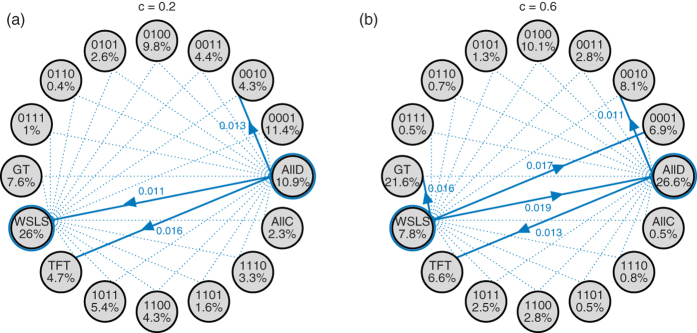
Evolutionary dynamics in the space of deterministic memory-one strategies, 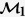 for two different cooperation costs. As in Fig. 1a, the grey circles correspond to all possible homogeneous populations, and blue lines indicate evolutionary transitions; for clarity, we only show transitions from *WSLS* or *AllD*. In (**a**), the cost of cooperation is sufficiently low such that *WSLS* is evolutionary robust. In (**b**), mutants using *AllD*, Grim Trigger *GT*, or the strategy (0, 0, 0, 1) can invade a *WSLS* population; as a consequence, *AllD* becomes most abundant in the selection-mutation equilibrium. Parameters are the same as in Fig. 1, population size *N* = 100, error rate *ε* = 0.01, and selection strength *w* = 0.1.

Let us next consider the space of deterministic memory-one strategies, which contains all 16 tuples of the form (*p*_*CC*_, *p*_*CD*_, *p*_*DC*_, *p*_*DD*_) with *p*_*ij*_ ∈ {0, 1}. Although the state space is now bigger, we can still apply the previous methods to calculate each strategy’s share in the selection-mutation equilibrium. Figure 2 illustrates two different parameter scenarios (both assuming an intermediate selection strength, *w* = 0.1). When the costs of cooperation are sufficiently low (Fig. 2a), the self-cooperating strategy *WSLS* is evolutionary robust: all other mutant strategies have a fixation probability smaller than 1/*N*. In contrast, a population of defectors is not robust: *AllD* is susceptible to invasion by *TFT*, *WSLS*, or by the strategy (0, 0, 1, 0). As a consequence, *WSLS* is the strategy that is most frequently used over time—in the invariant distribution, the share of *WSLS* is 26.0%, whereas the share of *AllD* is only 10.9%.

**Figure 3:**
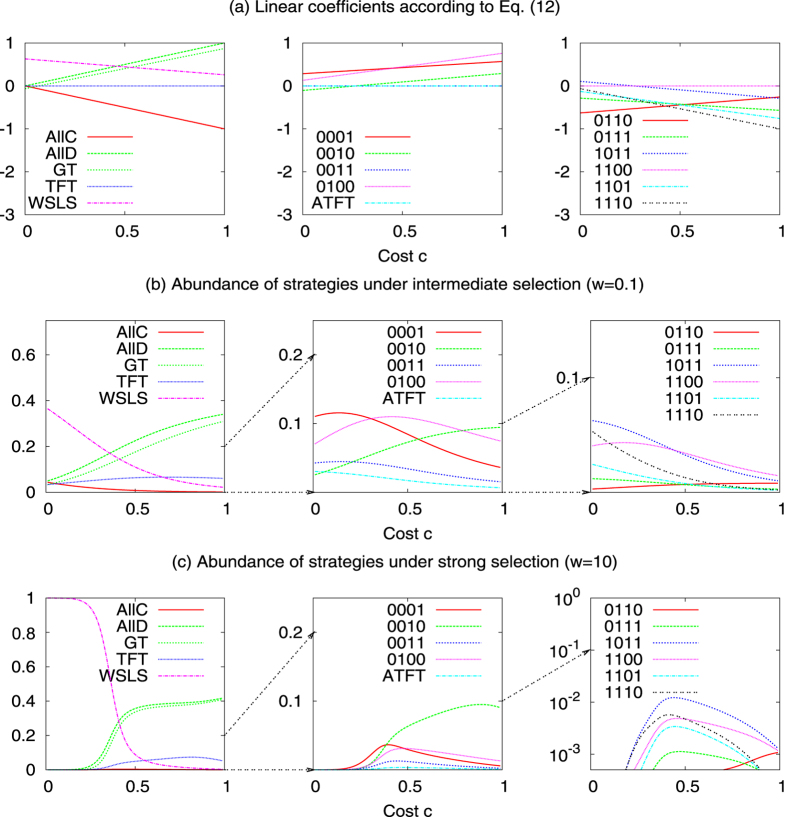
Selection-mutation equilibrium in the space of memory-one strategies for different costs and selection strengths. The graphs in (**a**) show the linear coefficients *L*_*i*_ according to Eq. (12), whereas the graphs in (**b,c**) show the strategy abundance for intermediate (*w* = 0.1) and strong (*w* = 10) selection, respectively. In each case, the 16 curves are plotted in three different panels (depending on the strategy’s abundance), in order to increase the clarity of the Figure. *WSLS* is most abundant when cooperation is cheap, whereas *AllD* and *GT* become predominant as *c* exceeds a critical threshold. The other parameters are the same as before, *N* = 100 and *ε* = 0.01.

The situation changes, however, when the cooperation costs exceed a critical threshold, as in Fig. 2b. In that case, *WSLS* ceases to be evolutionary robust. For example, in a homogeneous population of *WSLS* players, playing *WSLS* yields the mutual cooperation payoff 1, whereas playing *AllD* yields the temptation payoff 1 + *c* in one round and the mutual defection payoff *c* in every other round. Consequently, *AllD* receives the higher payoff whenever *c* > 1/2. Although *AllD* is not evolutionary robust either, it now obtains the largest share in the selection-mutation equilibrium (with 26.6%, as compared to the 7.8% of *WSLS*). Numerical calculations confirm that *AllD* becomes the most abundant strategy as the cost-to-benefit ratio approaches 1/2 (see Fig. 3). On the positive side, when cooperation is relatively cheap and when selection is strong, *WSLS* can reach almost 100% in the selection-mutation equilibrium (Fig. 3c).

Again, we can derive analytical results in the limit of weak selection by calculating the linear coefficients *L*_*i*_ according to Eq. (12). There are only a handful of strategies for which *L*_*i*_ > 0 independent of the value of *c* (see also Fig. 3a). Among these are *AllD* and *WSLS*,


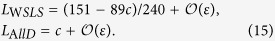


In particular, *WSLS* is most abundant when *L*_W*SLS*_ > *L*_*AllD*_, or equivalently, when 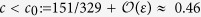.

### Strategy dynamics among the stochastic reactive strategies

**Figure 4:**
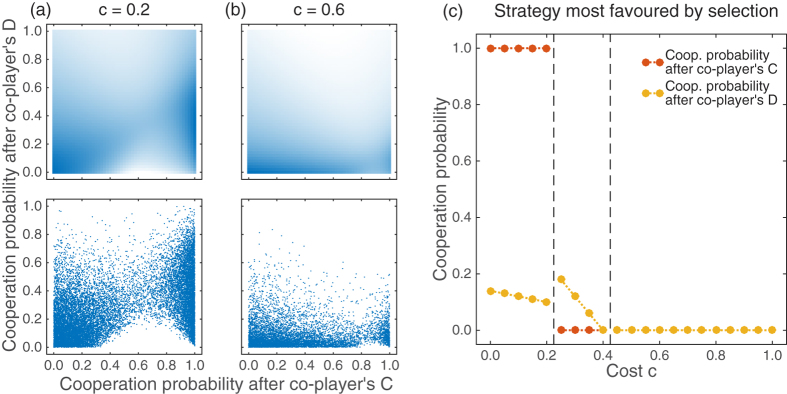
Evolutionary dynamics in the space of stochastic reactive strategies,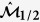. (**a,b**) illustrate our approximation for the invariant distribution for two different cost values, *c* = 0.2 and *c* = 0.6. For the upper graphs, we have calculated the invariant distribution for the discretised state space, where the conditional cooperation probabilities of the reactive strategy are taken from the (finite) set {0, *δ*, 2*δ*, …, 1 − *δ*, 1}, using a grid size *δ* = 0.02. Areas in dark blue colour correspond to strategy regions that have a relatively high frequency in the invariant distribution. The lower graphs show the results of simulations for the Imhof-Nowak process^51^; each blue dot represents a strategy adopted by the resident population. Both methods confirm that when the cost of cooperation is small, e.g. *c* = 0.2, the resident strategies are either clustered around the lower left corner or around the right edge of the state space. As the cost increases, more weight is given to the lower edge. In (**c**) we show the strategy that is most favoured in the limit of weak selection, i.e., the strategy with the highest linear coefficient *L*(**p**) according to Eq. (13). The graph indicates that there are three parameter regions: for low cost values, a generous strategy is most favoured; for intermediate cost values, the most favoured strategy has only a positive cooperation probability if the co-player defected previously; and for high cooperation costs *AllD* is most favoured. Parameters: Population size *N* = 100, *ε* = 0.01, and *w* = 10; the Imhof-Nowak process was simulated over 5 · 10^6^ mutant strategies.

Let us next turn to stochastic reactive strategies. In that case, players only pay attention to the co-player’s previous move (i.e., *p*_*CC*_ = *p*_*DC*_ and *p*_*CD*_ = *p*_*DD*_), but now they are able to choose their cooperation probabilities from the unit interval, *p*_*ij*_ ∈ [0, 1]. In particular, there are now infinitely many feasible strategies, which renders a full calculation of all transitions between possible homogeneous populations impossible. To cope with this issue, we have used two numerical approximations. The first method approximates the infinite state space by a finite grid (to which the previously used methods for finite strategy spaces can be applied). For two different cost values, we have illustrated the resulting invariant distribution in the upper panels of Fig. 4a,b. Figure 4a indicates that when cooperation costs are low, there are two strategy regions with a high abundance according to the invariant distribution. The first region corresponds to a neighbourhood of *AllD* (i.e. strategies for which both conditional cooperation probabilities are low); the second region comprises a set of generous strategies. In that region, players always reciprocate their opponent’s cooperation, while still exhibiting some degree of forgiveness in case the opponent defected in the previous round. However, as the cooperation costs increase (as in Fig. 4b for which *c* = 0.6), the region of generous strategies is visited less often, and defective strategies become predominant.

We obtain a similar result when we use our second method to approximate the dynamics within the space of stochastic reactive strategies. For this method, we have applied the dynamics of Imhof & Nowak^51^: starting from a population of defectors, we have repeatedly introduced single mutants into the population, who may adopt an arbitrary stochastic strategy (i.e., this time, strategies are not restricted to some finite grid). The mutant strategy may then either fixate or go extinct, leading to a sequence of resident populations over time. The lower panels in 4a and 4b depict the residents in this sequence as blue dots (for clarity, we have only plotted those resident populations that survived at least 50 mutant invasions). Again, low cooperation costs lead to two clusters in the two-dimensional state space—a cluster with defective strategies and a cluster with generous strategies. But as before, the cluster of generous strategies tends to shrink as the cooperation costs increase (as also observed in ref. 51). We have also numerically computed the stochastic reactive strategy that is most favoured by selection (see Fig. 4c). There are three parameter regions: for cost-to-benefit ratios below 1/4, we observe that the most favoured strategy is generous. However, as the cooperation costs increase and the cost-to-benefit ratio is between 1/4 and 2/5, the most favoured strategy prescribes that players should no longer reciprocate cooperation, and players should only cooperate with some low probability when the opponent defected in the previous round. Clearly, a population made up of such players only achieves low levels of cooperation. The situation becomes even worse as the cost-to-benefit ratio exceeds 2/5, in which case unconditional defection becomes the most favoured strategy.

### Strategy dynamics among the stochastic memory-one strategies

**Figure 5:**
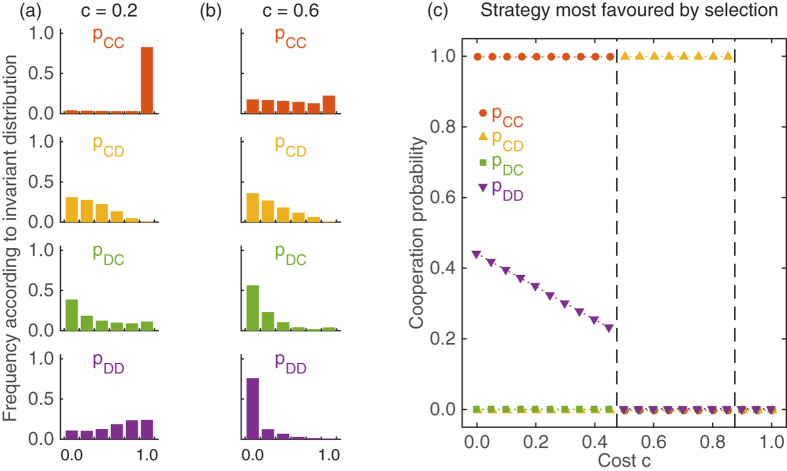
Evolutionary dynamics in the space of stochastic memory-one strategies,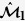. (**a,b**) show the marginal distribution of the evolving cooperation probabilities *p*_*ij*_ in the mutation-selection equilibrium. To generate the figure, we have calculated the invariant distribution for a discretised version of the state space, using a grid size of *δ* = 0.2. For low costs, the cooperation probabilities are in line with *WSLS* behaviour; for larger cost values, cooperation breaks down, and most evolving strategies are self-defectors. In (**c**) we depict the strategy that has the highest linear coefficient *L*(**p**) according to Eq. (13). Again there are three parameter regions: for low costs, a variant of *WSLS* is most favoured by selection; for intermediate costs, the somewhat paradoxical strategy (0, 1, 0, 0) is most favoured; and for high costs, *AllD* becomes predominant. Parameters are the same as before: Population size *N* = 100, *ε* = 0.01, and *w* = 10.

Finally, we can apply the same two approximations to the 4-dimensional space of all stochastic memory-one strategies. Of course, that state space can no longer be depicted in a two-dimensional graph; but Fig. 5a,b show the invariant distribution for each of the four components *p*_*CC*_, *p*_*CD*_, *p*_*DC*_, and *p*_*DD*_, again for the two cost values *c* = 0.2 and *c* = 0.6. For *c* = 0.2 we observe behaviour that is consistent with *WSLS*. After mutual cooperation, players almost certainly continue with cooperation, and after mutual defection players are more likely to cooperate than to defect, whereas the values of *p*_*CD*_ and *p*_*DC*_ rather prescribe to defect in the next round. On the other hand, when *c* = 0.6, the invariant distribution shows a bias towards self-defector strategies, as mutual defection in one round is most likely to lead to mutual defection in the next round. Again, we have also calculated the strategy most favoured by selection in the limit of weak selection (Fig. 5c). As in the case of stochastic reactive strategies, there are three scenarios: a cooperative scenario in which the population applies a variant of *WSLS* when cooperation costs are low; an intermediately cooperative scenario where the population uses the strategy p^*^ = (0, 1, 0, 0); and a defection scenario of an *AllD* population when cooperation costs are high. Compared to the case of reactive strategies, the fully cooperative strategy is now favoured for a wider range of cost values—the *WSLS* variant is most abundant for costs *c* ≲ 0.45, whereas the *GTFT*-like strategy depicted in Fig. 4c can only succeed when *c* ≲ 0.25. *WSLS* variants of the form (1, 0, 0, *x*) have the advantage of being immune against the invasion by both, *AllC* and *AllD* mutants (provided that *x* is sufficiently small for given cooperation costs). However, as opposed to the pure WSLS strategy (1, 0, 0, 1), strategies of the form (1, 0, 0, *x*) with *x* < 1 are not evolutionary robust. In the presence of errors, they can be invaded by strategies that yield a better approximation to *WSLS*, (1, 0, 0, y) with *y* > *x*, which in turn are more susceptible to invasion by *AllD*. As a consequence, we observe that the parameter region for which *WSLS* variants are most favoured in the space of stochastic memory-one strategies is comparable to the region for which the pure *WSLS* strategy is most abundant among the deterministic strategies (as depicted in Fig. 3).

Among the strategies most favoured by selection, the strategy p^*^ = (0, 1, 0, 0) comes most unexpected^58^. This strategy prescribes to cooperate only if one has been exploited in the previous round—which seems to be a rather paradoxical response. For small errors, a homogeneous population of p^*^ players yields an expected payoff of π^*^ = (1 + 3*c*)/4; two p^*^-players would typically defect against each other, but if one of the player cooperates by error, there can be long periods of unilateral cooperation. However, a single mutant applying *AllD* obtains the higher payoff (1 + 3*c*)/3, and thus one would expect that homogeneous p^*^ populations quickly disappear. But if p^*^ is not evolutionary robust, how can it be most favoured by selection for intermediate cost ranges?

Although *AllD* could easily invade a p^*^–population, it is highly unlikely that within the space of stochastic memory-one strategies the next mutant actually adopts *AllD*. Instead, most arising mutants would use strategies **p** = (*p*_*CC*_, *p*_*CD*_, *p*_*DC*_, *p*_*DD*_) for which all cooperation probabilities *p*_*ij*_ are strictly positive. In the limit of small errors, *ε* → 0, the payoff of such mutants in a p^*^–population can be computed as


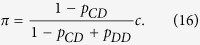


This payoff is not only smaller than the residents’ payoff π^*^; it is exactly the same payoff that mutants would get in an *AllD* population. Thus, the strategy p^*^ = (0, 1, 0, 0) can be successful because against almost all mutant strategies it behaves like *AllD*; only against itself (and against a few other strategies, like against *AllD*) it cooperates occasionally. In a sense, p^*^ acts as if it used a rudimentary form of kin recognition - it shows some cooperation against players of the same kind, but it defects against almost everyone else.

### Comparison of the evolving cooperation rates

**Figure 6:**
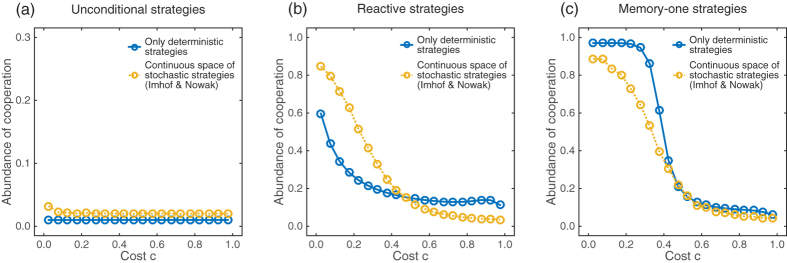
Evolving cooperation rates for (**a**) unconditional strategies, (i.e., strategies that use the same cooperation probability *p* in every round, independent of the past history), (**b**) reactive strategies, and (**c**) memory-one strategies. All graphs show the abundance of cooperation as measured by the quantity *γ* in Eq. (11) for the case of deterministic strategies (blue), and according to the Imhof-Nowak process for stochastic strategies (yellow; a discretised version of the continuous space of memory-one strategies would yield similar results). Dots represent simulation results, whereas solid lines represent numerically exact results derived from the invariant distribution of the evolutionary processes. Parameters: population size *N* = 100, *ε* = 0.01, and *w* = 10.

After analysing the strategy dynamics in each of the four strategy spaces separately, we are now in a position to compare the evolving cooperation rates. For reactive strategies and low cooperation costs, stochastic strategies lead to more cooperation than deterministic strategies (Fig. 6b). As we have seen in Fig. 1, deterministic reactive strategies are unable to stabilize cooperation; *TFT* can be invaded by *AllC*, and *AllC* is easily invaded by *AllD* (see also ref. 59). Stochastic reactive strategies, on the other hand, can maintain a healthy level of cooperation for a considerable time. *GTFT*-like strategies resist invasion by *AllD*, and they are only destabilized when altruistic *AllC*-like strategies increase in frequency by neutral drift^42^,^51^,^60^,^61^,^62^,^63^. However, with increasing cooperation costs, it takes longer until *GTFT*-like strategies emerge, as the so-called cooperation-rewarding zone shrinks as *c* increases (see, for example, ref. 5), and *GTFT*-like strategies are more likely to be invaded by overly altruistic strategies. As a result, when cooperation costs are high deterministic strategies perform slightly better, because *TFT* mutants show up more quickly to re-invade *AllD* populations.

Memory-one strategies are generally more favourable to cooperation, as depicted in Fig. 6c. In contrast to reactive strategies, memory-one strategies allow for *WSLS*-like behaviour which is more stable against indirect invasion by altruistic *AllC* strategies^44^,^64^. Interestingly, however, we find that for low cooperation costs, deterministic memory-one strategies are better in sustaining cooperation than stochastic strategies. Among the deterministic memory-one strategies, mutants are strongly opposed by selection when they enter a *WSLS* population (as illustrated in Fig. 2). As a result, *WSLS* reaches almost 100% in the invariant distribution, provided that selection is sufficiently strong and that the costs of cooperation are low. There are two reasons why stochastic strategies can result in less cooperation. First, although *WSLS* remains a Nash equilibrium^65^,^66^, stochasticity allows for the invasion of nearby mutants (that are only slightly disfavoured by selection); these mutants may in turn be more susceptible to invasion by *AllD*^67^. Second, stochastic dynamics often generates resident populations that only use an approximate version of *WSLS*, having the form (1, 0, 0, *x*), with *x* < 1. Compared to the deterministic *WSLS* rule, these approximate versions are more prone to noise: if one of the players defected by error, it may take a substantial number of rounds to re-establish mutual cooperation (which becomes most clear when *x* is close to zero).

**Figure 7:**
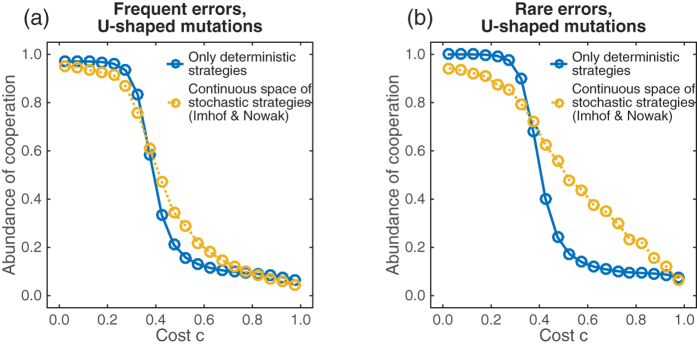
U-shaped mutation kernels lead to more cooperation in high cost scenarios. As in Fig. 6c, both graphs show the evolving cooperation rate for the space of deterministic memory-one strategies (blue) and stochastic memory-one strategies (yellow). However, here we have varied the error rate of players (*ε* = 1% for frequent errors, *ε* = 0.01% for rare errors). In addition, the cooperation probabilities *p*_*i*_ of new mutant strategies are now taken from a beta-distribution. The beta-distribution has the density function *f*(*p*) = *Cp*^*α*−1^(1 − *p*)^*β*−1^, with *C* being a normalization factor. The values *α* = *β* = 1 yield the uniform distribution on [0,1], as used in Fig. 6; here, we have taken *α* = *β* = 0.1, yielding a strongly U-shaped distribution. All other parameters are the same as in Fig. 6.

This result is somewhat disappointing: especially in parameter regions in which *WSLS* is unstable, one would hope that stochastic strategies allow at least for some degree of cooperation, because WSLS variants of the form (1, 0, 0, *x*) are immune to the invasion of *AllC* and *AllD* mutants as explained above. The previous results on the effect of stochasticity need to be viewed in light of the assumed mutation kernel—for our numerical results we have assumed that new mutant strategies are taken from a uniform distribution. This assumption often generates mutant strategies with intermediate cooperation probabilities—which have no chance of being evolutionary robust^40^. What would happen if mutant strategies were instead taken from a distribution that puts more weight on the boundary of the state space? In Fig. 7, we show numerical results under the assumption that the cooperation probabilities of new mutant strategies follow a U-shaped distribution on the interval [0,1]. Keeping the previous error rate of *ε* = 0.01, the U-shaped mutation kernel seems to marginally increase the evolving cooperation rates for most cost values (Fig. 7a). If we additionally reduce the error rate to *ε* = 10^−4^, U-shaped mutations can lead to a more dramatic increase in cooperation rates, especially for scenarios with intermediate cooperation costs. In that parameter region, successful residents often apply strategies of the form (1 − *δ*_1_, *δ*_2_, *δ*_3_, *δ*_4_) with all *δ*_*i*_ ≪ 1. Because *δ*_4_ ≪ 1, such residents can hardly be exploited by *AllD* mutants. If, in addition, *δ*_1_ ≪ *δ*_4_, such strategies can still reach a substantial level of cooperation against themselves. We note that strategies of the form (1 − *δ*_1_, *δ*_2_, *δ*_3_, *δ*_4_) are not stable, as they could be invaded by strategies that increase their cooperation probability after mutual defection. However, provided that *δ*_1_ is sufficiently small, the selective advantage of such mutants would be comparably small, and hence it may take a long time until such mutant strategies appear and fixate in the population. The results in Fig. 7 thus suggest that the assumed mutation structure can have a considerable impact on the evolving cooperation rates. Herein, we have considered two extreme structures, uniform mutations and strongly U-shaped mutations, but a more general analysis of the impact of different mutation kernels would certainly be a worthwhile topic for future research.

## Discussion and Summary

We have used the Moran process in finite populations to study the evolution of cooperation in repeated games. The mathematics of repeated games can be intricate. Even if one only considers a restricted strategy space, such as the space of all memory-one strategies, it is typically hard to derive exact results for the resulting evolutionary dynamics. There are various ways to cope with this complexity. Some studies have focused on even simpler strategy sets, consisting only of a handful of representative strategies (e.g. refs 7,59,61 and 68). Others have obtained analytical results for certain infinitely-dimensional subsets of memory-one strategies, like reactive strategies^51^,^60^, zero-determinant strategies^62^, or conformistic strategies^63^. Yet another approach is to use computer simulations (as in refs 44 and 69, 70, 71). Herein, we have taken a somewhat intermediate approach. By assuming appropriate separation of time scales (e.g., mutations are sufficiently rare such that populations are typically homogeneous), we can compute numerically exact strategy abundance in case the strategy space is finite (as in the case of deterministic strategies). To explore the dynamics among stochastic strategies, we have extended this approach to approximate the dynamics in infinite strategy spaces.

We have used this approach to systematically compare the evolutionary dynamics among strategy spaces of different complexity. The strategy spaces considered differ along two dimensions, depending on whether strategies are reactive or memory-one, and depending on whether strategies are deterministic or stochastic. Each of the four considered strategy spaces has been explored previously, but only in isolation. Herein, we are explicitly interested how much complexity is needed to allow for a healthy level of cooperation. In this way, our study contributes to a growing research effort, exploring how the evolution of cooperation depends on underlying modelling assumptions. For example, García and Traulsen^72^ and Stewart and Plotkin^71^ have analysed the role of the mutation structure on the emergence and stability of cooperation, whereas van den Berg and Weissing^73^ have explored the consequences of two different strategy representations. We believe that this kind of research is extremely useful, as it serves as an important robustness check for previous results on the evolution of direct reciprocity.

Our study provides at least two major insights. The first insight is that more complex strategies do not guarantee more cooperation. More specifically, we have found that memory-one strategies, which also take one’s own previous move into account, have a positive impact on cooperation. If players have no memory at all (i.e. if they can only use unconditional strategies), evolution unambiguously promotes defection (as depicted in Fig. 6a). However, if players can react to the co-player’s previous move, or even better to the moves of both players, then evolution can promote cooperative strategies when the costs of cooperation are sufficiently low. Although we have not tested memory-two strategies (i.e. players who react to the outcome of the last two rounds), one may expect that such strategies could further facilitate cooperation, especially in parameter regions in which the classical *WSLS* strategy becomes unstable (see, e.g. refs 8 and 74). The effect of stochasticity on cooperation is more ambiguous. If players only remember the co-players’ previous move, then stochasticity allows for generous strategies like *GTFT*, and such generous strategies can help to establish relatively high levels of cooperation. On the other hand, when cooperation costs are low, and players are allowed to use memory-one strategies, stochastic strategies cannot further promote cooperation. Here, the deterministic version of *WSLS* works best.

Our second insight is rather conceptual. To quantify the evolutionary success of some strategy **p**, it is common to check whether the strategy is an equilibrium, or whether the strategy is evolutionary robust (see e.g. refs 40,50,65,66 and 75). To this end, one checks whether there would be a mutant strategy **q** that can prosper in a population of **p** players. A strategy that is not robust is generally assumed to play a minor role during the evolutionary process. Yet, we have seen that under some evolutionary conditions, the strategy p^*^ = (0, 1, 0, 0) can be surprisingly successful despite not being evolutionary robust. This somewhat paradoxical strategy can persist because against almost all other strategies it plays like *AllD*; but against a handful of strategies (including itself and against *AllD*) it cooperates for a substantial fraction of time. In particular, there are mutant strategies that could invade into a homogeneous p^*^ - population. However, the probability that such a mutant arises within a reasonable timespan is vanishingly small, as the space of such advantageous mutants has measure zero within the space of all memory-one strategies. Thus, it does not seem sufficient for evolutionary robustness to ask whether there is another strategy that would have a higher fitness; one also needs to check whether this beneficial mutant strategy can arise under the considered mutation scheme. Put differently, unless a resident outperforms every other strategy, the question of evolutionary robustness cannot be properly assessed without reference to the mutation scheme. Of course, this observation does not diminish the value of traditional equilibrium considerations—but if a strategy is only unstable because some non-generic strategy can invade, then some caution seems warranted.

### Appendix: Computation of the linear coefficient *L*(p) for stochastic strategies

To compute the stochastic strategy that is most favoured by selection, we have evaluated the four-dimensional integral *L*(**p**) in Eq. (13) by means of Gaussian quadrature^76^. For maximizing *L*(**p**), we have employed a two-step approach: The first step is exhaustive global search of the whole strategy space. Some degree of discretisation is inevitable in checking many different realizations of **p** = (*p*_*CC*_, *p*_*CD*_, *p*_*DC*_, *p*_*DD*_). In particular, we have observed that the objective function *L*(**p**) tends to change rapidly when **i** approaches the boundary of the strategy space. As the change is smoothed by the implementation error, it is quite often the case that 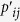, or 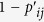, turns out to be 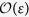. Therefore, the mesh size of *p*_*ij*_ has been set to be of an order of *ε* when getting close to zero or one. Specifically, we have used 17^4^ = 83, 521 grid points in total by adding *p*_*ij*_ = 0.005, 0.01, 0.02, 0.98, 0.99, and 0.995 to a regular mesh grid *p*_*ij*_ = 0.1*k* (*k* = 0,…, 10).

The next step is the gradient-descent method^77^, starting from the best strategy of the exhaustive search. Although this second method is local, it works in a continuous space and finds out a nearby maximum with far higher precision than the grid search. We expect that this two-step approach precisely locates the global maximum as long as the mesh of the first step is fine enough to detect all the relevant variations of the objective function *L*(**p**).

## Additional Information

**How to cite this article**: Baek, S. K. *et al*. Comparing reactive and memory-one strategies of direct reciprocity. *Sci. Rep*. **6**, 25676; doi: 10.1038/srep25676 (2016).

## Figures and Tables

**Table 1:** Four different strategy spaces considered in this work. Each parameter *p*_*ij*_ denotes the focal player’s probability to cooperate in the next round, given that the player’s previous action was *i* and that the co-player's action was *j*.

	Reactive strategies	Memory-1 strategies
Deterministic strategies	Deterministic reactive strategies, 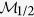 *p*_*CC*_ = *p*_*DC*_, *p*_*CD*_ = *p*_*DD*_ *p*_*ij*_ ∈ {0, 1}	Deterministic memory-1 strategies, 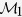 *p*_*ij*_ ∈ {0, 1}
Stochastic strategies	Stochastic reactive strategies, 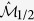 *p*_*CC*_ = *p*_*DC*_, *p*_*CD*_ = *p*_*DD*_ *p*_*ij*_ ∈ [0, 1]	Stochastic memory-1 strategies, 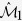 *p*_*ij*_ ∈ [0, 1]
